# Personal

**Published:** 1903-12

**Authors:** 


### PERSONAL.

Dr. Edward C. Mann, of Buffalo, having finished his term of service as interne at the General Hospital, sailed for Europe early in November. He will remain during the winter in Berlin, and before returning in the early summer, will spend some time at each of the principal medical centers of Europe, further pursuing medical research.

Dr. John R. Gray, of Buffalo, who last spring removed from 246 Seventh Street to his new residence and office at 423 Prospect Avenue, is preparing one of the most extensive floral gardens in the city. He has over two thousand bulbs under cultivation and during the next season expects to make a handsome display of bulbous and other flora.

Dr. H. M. Edmonds, of Tonawanda, has been appointed health officer of that city, vice Dr. R. C. Taber, resigned.

Dr. E. L. Shurly, of Detroit, recently visited Buffalo, and attended the annual dinner of the Alumni of Buffalo General Hospital, which was given at The Touraine, Saturday evening, November 7, 1903. Dr. Shurly's humorous speech was well received by the assembly.

